# The anaerobic digestion microbiome is robust toward variation in the waste activated sludge feed

**DOI:** 10.1093/ismeco/ycaf072

**Published:** 2025-04-25

**Authors:** Josefien Van Landuyt, Jasmine Oosterlinck, Jo De Vrieze

**Affiliations:** Centre for Microbial Ecology and Technology, Universiteit Gent, Frieda Saeysstraat 1, Gent B-9052, Belgium; Centre for Microbial Ecology and Technology, Universiteit Gent, Frieda Saeysstraat 1, Gent B-9052, Belgium; Witteveen+Bos Belgium N.V., Posthoflei 5, Bus 1, 2600 Antwerpen—Berchem, Belgium; Centre for Microbial Ecology and Technology, Universiteit Gent, Frieda Saeysstraat 1, Gent B-9052, Belgium; Centre for Advanced Process Technology for Urban Resource Recovery, Frieda Saeysstraat 1, Gent B-9052, Belgium

**Keywords:** ecological microbiology, microbial community composition, seasonal fluctuations, anaerobic digestate quality, community analysis, 16S rRNA gene sequencing

## Abstract

Anaerobic digestion stands out as the foremost technology for maximizing the valorization of waste activated sludge (WAS) to recover energy and recover resources. The physical/chemical and microbial makeup of WAS is susceptible to seasonal fluctuations, due to the open-air nature of wastewater treatment facilities, potentially impacting subsequent digester performance and the quality of the resulting digestate. This study delved into a comprehensive analysis of both the initial WAS and the digestate produced by 12 full-scale digesters during both a summer and winter sampling campaign. A significant influence of seasonal variations was observed on the physical/chemical and microbial composition of WAS. Interestingly, the digestate microbiome exhibited a high resilience with minimal seasonal fluctuations, but instead showed variations between different digesters. In summary, this research demonstrates that while WAS composition manifests in specific physical/chemical attributes, it does not exert a discernible influence on the microbial composition of the resulting digestate.

## Introduction

In the current anthropocentric world undergoing rapid climate change, resource scarcity is driving a paradigm shift for waste management from dissipative treatment to resource recovery. The circular economy concept entails that beyond clean water recovery from wastewater management, valuable materials should be extracted from wastewater [1]. Energy derived from wastewater sludge may be a sustainable solution to meet current human energy requirements [2]. One of the most established circular technologies in wastewater sludge treatment is anaerobic digestion (AD). The AD process has been optimized over the last century, transforming it into a mature technology allowing for the conversion of diverse organic waste streams into biogas, a renewable source of methane (CH_4_) [3]. The remaining sludge (digestate) is a nutrient-rich organic residue that can be processed further for various applications. It can be used directly as fertilizer in agriculture, composted, or, more recently, subjected to pyrolysis to produce biochar [4, 5].

Commonly observed hydrolytic and acidogenic bacteria in AD systems are often members of the Proteobacteria (Pseudomonadota), Firmicutes (Bacillota), or Bacteroidetes (Bacteroidota) phyla [6, 7]. The abundance and ratio of the common key players in the anaerobic microbiome have been linked to process performance [8]. The ratio of Bacteroidota to Firmicutes*,* for example, which has also been identified as a relevant marker in human gut health [9, 10], has been shown to be correlated with methane production and overall digester health [8]. Cayetano *et al.* [11] showed that a Bacteroidota to Firmicutes ratio of 4 or higher could be correlated with a significant decrease in methane production in both batch and continuous laboratory digesters. Part of the high abundance AD microbiome seems to be non-growing and possibly inactive immigrating microbes. These are introduced into the digester from the feed (e.g. waste activated sludge (WAS) microbiome), suggesting that the feedstock microbiome could have a significant effect on the digester microbiome composition [12]. However, this non-growing biomass could also offer functional redundancy and resilience (defined in Allison and Martiny [13]) when facing process disturbances. De Vrieze *et al.* [14] found that the digester microbiome was able to fully recover its function (i.e. methane production) after salt stress, due to the functional redundancy in the microbial community. Operational parameters, such as incoming organic content [chemical oxygen demand (COD) of the feed], pH, temperature, and sludge retention time can influence the microbial composition, and this, in turn, influences the productivity of the anaerobic digester (AD). For example, the volatile solids (VS) to total solids (TS) ratio of the feed was shown to influence the microbial composition, with an increasing VS/TS related to an increasing ratio of archaea to bacteria, resulting in an increased methane production [15]. An alternative example is an incoming stream high in nitrogen-rich substrates, which can lead to high total ammonia concentrations, sometimes resulting in a shift from acetoclastic methanogenesis to syntrophic acetate oxidation coupled with hydrogenotrophic methanogenesis [16].

Seasonality of the incoming feedstock during AD of, for example, food waste shows a significant effect on the methane productivity [17] and the microbial composition [18]. Composition of food waste can be highly seasonally dependent, as food crops are seasonal, whereas the composition of WAS appears less seasonal. However, wastewater treatment facilities are open air and, thus, subject to climatic influences (e.g. temperature and rainfall). The seasonality of the incoming wastewater streams and their subsequent effect on the activated sludge community can alter the community significantly [19]. How seasonal changes in the microbial composition of the WAS and potentially in the physicochemical parameters of WAS affect the microbial composition of sludge treatment processes, such as AD, could be of clear interest for the improvement of the digester process control.

In this study we performed an in-depth microbial (taxonomy-based) and physical/chemical analysis of both WAS feedstock and digestate to identify the effect of seasonal differences on relevant process parameters [VS, TS, ammonium, volatile fatty acids (VFA), pH, and salinity] and the microbial composition of WAS and the anaerobic digestate. We hypothesize that industrial-scale anaerobic sludge digester microbiomes have a certain robustness regardless of the incoming feed chemistry and microbial community and that the inter-variability between different digesters contributes more significantly to the diversity between microbiomes than the effect of seasonality.

## Materials and methods

### Sample and data collection—in-depth description

**Figure 1:**
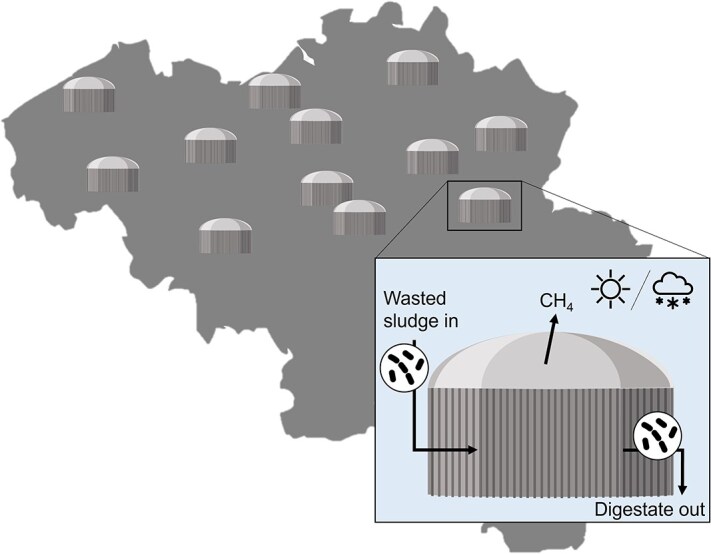
Graphical representation of the 12 different AD sampled at the entrance [waste activated sludge (WAS) going in] and the outgoing stream (digestate) across Flanders (map of Belgium downloaded from vecteezy.com).

Twelve different wastewater treatment plants (WWTP) in Flanders, operated by Aquafin NV, were sampled. A sample (*n* = 1) was taken at two different timepoints from both the (aerobic) WAS being fed to the AD, as well as the (anaerobic) digestate from inside the digester (Fig. 1). Digesters were all fed with non-pretreated secondary sludge (wasted activated sludge). The samples were taken both in summer and late autumn/early winter (19–21 August 2019; 10–12 December 2019). For confidentiality reasons, the WWTP facilities are anonymously numbered from 1 to 12. The same facilities were used in the summer and winter sampling campaign. Samples were aliquoted and partly stored in the fridge at 4 ± 1°C until further chemical analyses and experiments could be performed. The remainder was immediately frozen and stored at −20°C for subsequent VFA and molecular analyses.

### Microbial community analyses: Illumina sequencing

For DNA extraction, a bead beating with a PowerLyzer (Qiagen, Venlo, the Netherlands) and phenol/chloroform extraction protocol was used, as described in De Paepe *et al.* [20]. After confirming the purity of the DNA through PCR and gel electrophoresis, extracts were sent to LGC genomics GmbH (Berlin, Germany) for library preparation and amplicon sequencing on an Illumina MiSeq platform (V3 chemistry), using the modified version of the 341F (5’-CCTACGGGNGGCWGCAG-3′) and 785R (5’-GACTACHVGGGTATCTAAKCC-3′) primers derived from Klindworth *et al.* [21].

### Analytical techniques

#### Total solids and volatile solids

The TS and VS of the WAS and digestate samples were determined according to the standard method 2540G ([22]) using three technical replicates for each digester. Small containers were heated in a muffle furnace at 550°C for at least an hour after which they were placed back in the desiccator overnight. Once containers cooled to room temperature in a desiccator, the mass of the empty containers was determined (A). Approximately 25 g of sample (WAS and digestate) was added to containers for mass determination (B). These samples were dried in an oven at 103–105°C overnight. The containers with dried WAS or digestate were consequently decomposed at 550°C in the muffle oven for 1.5 h. After cooling down in the desiccator, the mass was redetermined (D). The TS and VS were determined based on the following formulas.


(1.1) \begin{equation*} \%\mathrm{TS}=\frac{\left(\mathbf{C}-\mathbf{A}\right)\mathrm{x}100}{\mathbf{B}-\mathbf{A}} \end{equation*}

(1.2) \begin{equation*} \%\mathrm{VS}=\frac{\left(\mathbf{C}-\mathbf{D}\right)\mathrm{x}100}{\mathbf{C}-\mathbf{A}} \end{equation*}

With A = mass of empty container (g); B = mass of container + WAS or digestate (g); C = mass of container + dried mass before muffle (g); D = mass of container + ashes after muffle (g).

#### pH

The pH of the WAS and digestate samples were measured in triplicate using a Metrohm 744 pH measurer. The pH probe is calibrated weekly using three buffer solutions with a pH of 4.0, 7.0 and 9.0 respectively.

#### Conductivity

The conductivity of the WAS and digestate samples was measured in triplicate using a Consort C6010 conductivity measurer. The probe is calibrated weekly using three solutions with concentrations of 1, 0.1 and 0.01 M KCl (Chemlab-Analytical, Belgium).

#### Volatile fatty acids

The amount of VFA (C2–C8 fatty acids) present in the WAS and digestate samples were determined in triplicate by a diethyl ether extraction and measured by gas chromatography (GC-2014, Shimadzu ®, Nederland) with a DB-FFAP 123–3232 column (30 mm × 0.32 mm × 0.25 μm; Agilent, Belgium) and a flame ionization detector (FID), as described by Andersen *et al.* [23]. In short, the WAS or digestate sample were conditioned with sulfuric acid and sodium chloride and 2-methyl hexanoic acid as internal standard for quantification of further extraction with diethyl ether. Prepared ether extracts were subsequently injected in the GC-FID, which was calibrated for a VFA concentration range of 30–1000 mg/L for acetate, 10–1000 mg/L for propionate and for other VFA 2–1000 mg/L using a nitrogen gas carrier and the same parameters mentioned in Andersen *et al.* [23].

#### Cation IC

The concentrations of cations (Na^+^, NH_4_^+^, K^+^, Ca^2+^ and Mg^2+^) were determined in triplicate for each WAS and digestate sample. The analysis of the cations was performed using ion exchange chromatography (IC). Supernatants were centrifuged at 17090 × g and then filtered through a 0.2 μm filter diluted using Milli-Q water. The samples were analysed on a 761 compact ion chromatograph (Metrohm, Switserland) equipped with a conductivity detector. The cation IC machine had a detection range of 2–100 mg.L^−1^.

### Statistical analysis

#### Microbial data analysis

The amplicon sequence data were processed using the DADA2 (v1.26) package, according to the pipeline tutorial [24]. The detailed description of amplicon sequencing, as well as amplicon sequencing data processing are described in a previous work (Van [25]). In this work, both the Silva138 [26] and the MiDAS [27] databases were used for taxonomy assignment. For downstream filtering, amplicon sequence variants (ASVs) that were assigned a different taxonomy than “Bacteria” were removed from the data set, since bacterial primers were used to amplify and sequence the samples. To screen for the most abundant archaea in the samples, a subset archaeal ASV table was built for all digester samples. Statistical data analysis was performed in R (v4.2.1, [28]) using the “phyloseq” (for microbiome data handling) [29], “dplyr” [30], “vegan” (for diversity analysis) [31] and “deseq2” (for differential abundance analysis) [32] packages. Alpha- and beta-diversity metrics were calculated based on the first three (q = 0,1,2) Hill numbers [33] and the Bray–Curtis dissimilarity index [34], respectively. Significance (*P* < .05) was always tested either using parametric (paired) *t*-tests when the assumptions for a parametric test were met or non-parametric Wilcoxon-rank-sum tests. Permutational multivariate analysis of variance (PERMANOVA) was performed on the distance matrices to calculate significant grouping in beta-diversity when homogeneity of the variance was assured (through “*betadisper*” for analysis of multivariate homogeneity of group dispersions and “*permutest*” to test significance of this multivariate homogeneity of group dispersions). For the comparison between groups on the compositional level, Analysis of Compositions of Microbiomes with Bias Correction (ANCOMB-C) was applied [35].

Lists were assembled with all genera known to be nitrifiers and denitrifiers (see Supplementary Table S1) and all ASVs belonging to genera sharing this functionality in our samples were selected. Similarly to the nitrifiers/denitrifiers, lists were assembled of all genera known to be a part of the acidogen or acetogen functionality group; ASVs classified as these genera were selected (see Supplementary Table S1).

#### Chemical data analysis

Data analysis was performed in R (v4.2.1, [28]). Significance was always tested either using a parametric (paired) *t*-test when the assumptions for a parametric test were met (homoscedasticity and normal distribution of the data) or a non-parametric Wilcoxon-rank-sum test was used. Statistical Principle component analysis testing was performed using the “*PCAtest*” package [36]. Error propagation was used if applicable. In all cases, significance was assumed if the calculated *P*-value was below .05.

### Data submission

The fastq files obtained from this study were submitted to the National Center for Biotechnology Information (NCBI) under the Accession number PRJNA1144789 (reviewer link: https://dataview.ncbi.nlm.nih.gov/object/PRJNA1144789?reviewer=remo96iv4uol1159b0vt79st1r). The script to go from raw sequencing data to phyloseq objects as well as the in silico script to test the primers were uploaded on Github (reviewer link: https://github.com/jwvlandu/SeasonalVariationInAD).

## Results

### Physicochemical parameters: focus on differences between seasons

#### Wasted sludge samples show clear effect of season

**Figure 2:**
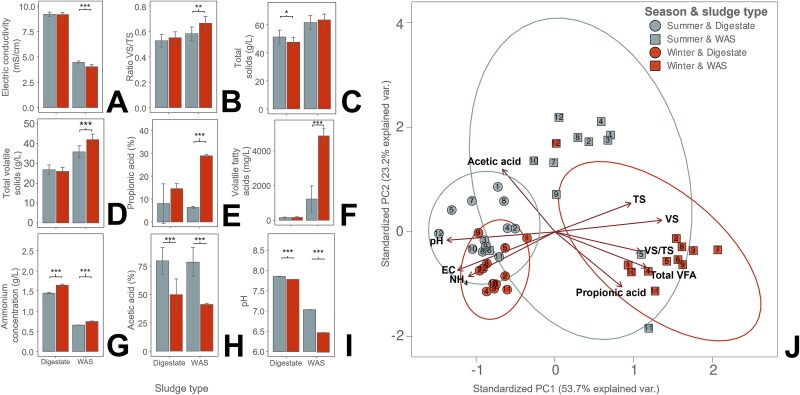
Average values of the different parameters per season and sludge type with significance calculated [error bars depict standard deviation (technical *n* = 3)], winter samples are the right sided bars (red), summer samples the left sided (blue) (**A**) EC (**B**) VS/TS (**C**) TS (**D**) Total VS (**E**) Propionic acid (**F**) Total VFA (**G**) Ammonium (NH_4_) concentration (**H**) Acetic acid and (**I**) pH, (*t*-test or Kruskal–Wallis test, “^*^” .05 > *P* > .01, “^*^^*^” .01 > *P* > .001, “^*^^*^^*^” .001 > *P*). (**J**) PCA of all parameters measured for all samples (both waste activated sludge (WAS) as well as digestate) with 95% confidence ellipses (Table S6).

The incoming stream of the AD always consisted of WAS; the measured chemical parameters are summarized in Table 1 and denoted for all samples separately in supplementary Tables S2 and S3. Notably, averages for samples taken in the summer were significantly different from the samples taken in winter (*t*-test, *P*-value <.05), except regarding TS concentrations (Fig. 2A–I and Table 1).

**Table 1:** Chemical parameters of the samples taken at the incoming stream (= feed) of the twelve AD treating WAS.

Parameter	Summer average	Winter average	*P*-value (winter vs. summer)	Minimum value	Maximum value
pH (−)	7.03 ± 0.01	6.46 ± 0.01	0.00001^*^	5.67 ± 0.08	7.70 ± 0.32
TS (g/L)	61.7 ± 1.7	63.5 ± 1.4	0.18	44.8 ± 30.2	81.6 ± 5.1
VS (g/L)	35.9 ± 3.0	42.0 ± 2.8	0.00011^*^	26.8 ± 0.7	58.0 ± 0.0
Conductivity (mS/cm)	4.45 ± 0.05	4.03 ± 0.09	0.00005^*^	2.16 ± 0.33	9.98 ± 1.04
NH_4_ (g/L)	0.65 ± 0.005	0.74 ± 0.01	0.00001^*^	0.19 ± 0.00	1.62 ± 0.02
Total VFA (mg/L)	1219 ± 753	4855 ± 446	0.00001^*^	34 ± 3	9179 ± 8784
Propionic acid (%)	6.3 ± 0.5	29.9 ± 0.5	0.00001^*^	0 ± 0	45.5 ± 1.3
Acetic acid (%)	78.6 ± 12.8	41.2 ± 1.2	0.00001^*^	2.1 ± 1.4	100 ± 0

Ammonium (NH_4_) (technical *n* = 3).

^*^Significant *P*-value

The measured chemical parameters of the digestate itself were summarized in Table 2 and denoted for all samples separately in supplementary Tables S4 and S5. However, in contrast with the wasted sludge parameters, not all chemical parameters measured were significantly (*t*-test; *P* < .05) different across seasons. Electric conductivity as well as certain suspended solids parameters and volatile fatty acid measurements were not significantly different (EC, VS/TS, total VFA content, propionic acid percentage and VS) between winter and summer. The other chemical parameters measured (TS, pH, ammonium, and acetic acid percentage) were significantly different (Fig. 2A–I).

**Table 2:** Chemical parameters of the samples taken of the digestate of AD treating WAS.

Parameter	Summer average	Winter average	*P*-value (winter vs. summer)	Minimum value	Maximum value	
pH (−)	7.85 ± 0.00	7.78 ± 0.01	0.00001^*^	7.52 ± 0.02	8.22 ± 0.05
TS (g/L)	51.3 ± 4.9	47.5 ± 3.6	0.02668^*^	32.7 ± 27.1	60.4 ± 2.3
VS (g/L)	26.8 ± 2.4	26.0 ± 2.1	0.21085	19.0 ± 8.9	32.2 ± 14.6
Conductivity (mS/cm)	9.18 ± 0.23	9.17 ± 0.21	0.47826	7.25 ± 0.27	12.34 ± 1.24
NH_4_ (g/L)	1.45 ± 0.02	1.64 ± 0.03	0.00001^*^	1.07 ± 0.39	2.15 ± 0.65
Total VFA (mg/L)	146 ± 39	162 ± 42	0.160314	29 ± 42	334 ± 399
Propionic acid (%)	8 ± 9	14 ± 2	0.01318^*^	0 ± 0	28 ± 3
Acetic acid (%)	80 ± 12	50 ± 14	0.00005^*^	33 ± 118	100 ± 0

^*^Significant *P*-value.

The principal component analysis (PCA) (Fig. 2J) for the measured physicochemical parameters showed noticeable grouping based on both sludge type (mainly x-axis) and sampling season (mainly y-axis). Permutation-based statistics showed the first two principal component (PC) axes as significant (*P* = 0), explaining 76.8% of the total variation. All variables had a significant loading on the first PC axis. The acetic acid and propionic acid variables had a significant loading on the second PC axis (Fig. 2J). The percentage of acetic acid and propionic acid were juxtaposed over the sampling season, while (total) ammonium concentration and TS best explained the difference between sludge types. Some grouping is observed (95% confidence ellipses on Fig. 2J) based on season for digestate samples despite temperature stability within the digester (mesophilic 34–37°C), as well as process parameter stability such as sludge retention time (SRT = 18 days), feeding times (continuous feeding), *etc*.

### Microbial community analysis

#### Community composition

**Figure 3:**
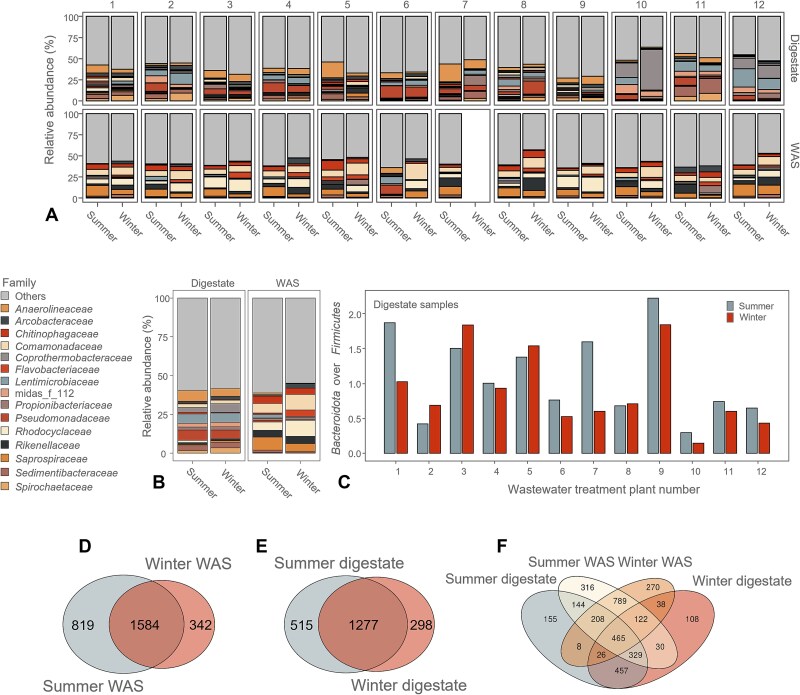
(A) The top 15 bacterial families are depicted in bar graphs, divided into separate graphs per wastewater treatment plant and per sludge type (waste activated sludge (WAS) or digestate). B. The top 15 bacterial families are depicted in bar graphs per condition (sludge and season). (C) Bar graph of the ratio of Bacteroidota to Firmicutes colored by season for each wastewater treatment plant. (D) Venn diagram visualizing the ASVs (amplicon sequence variant) shared by summer and winter WAS samples. (E) Venn diagram visualizing the shared ASVs of summer and winter digestate samples. (F) Venn diagram visualizing the shared ASVs across all four groups.

After comparison, the taxonomy assignment with the MiDAS database was chosen, as it was able to identify more ASVs (3465 vs. 2133) at the genus rank compared to the Silva138 database. The samples were dominated by Proteobacteria (digestate average: 15.43 ± 6.82% and WAS average: 29.17 ± 7.50%), Bacteroidota (digestate average: 20.44 ± 5.20% and WAS average: 34.61 ± 6.68%), Firmicutes (digestate average: 25.10 ± 9.68% and WAS average: 10.41 ± 6.94%) and Chloroflexi (digestate average: 8.49 ± 5.54% and WAS average: 2.21 ± 2.25%). Samples averaged out by sludge type and season (Fig. 3B) show that the bacterial community composition in the digestate samples did not differ across seasons (Bray–Curtis dissimilarity = 0.33) as much as the feed samples (WAS) (Bray–Curtis dissimilarity = 0.51).

A clear difference can be observed between the different sludge types regardless of season, with *Anaerolineaceae*, *Coprothermobacteraceae* and *Pseudomonadaceae* families abundantly present in the digestate and *Comamonadaceae*, *Chitinophagaceae* and *Rhodocyclaceae* families more abundantly presented in the WAS. The ratio *Bacteroidota* over *Firmicutes* remained below 4 (min: 0.3 and max: 3.3) in all WWTP digesters, independent of the season sampled. Apart from WWTP 7 (x 2.66), the ratio did not increase/decrease more than double between winter and summer ratios (Fig. 3C).

When averaged out in Fig. 3B, the WAS samples appear similar across seasons, however, there are some clear differences in composition of the bacteria present in the WAS across wastewater treatment plants (Fig. 3A) when looking at individual digesters. The digestate samples differ across wastewater treatment plants despite similar averages across seasons (Fig. 3A and B). When comparing summer to winter digestate samples per digester (Fig. 3A), a different profile emerges. However, this does not directly translate in the shared ASVs (Fig. 3D and E), since shared ASVs for WAS account for about 58% of all ASVs found in the WAS samples, while for digestate these cover up to 61% of all ASVs found in digestate samples, and, thus, only slightly more ASVs are shared across seasons in the digestate samples than in the WAS samples. In total, 465 ASVs were shared across all conditions (summer, winter, digestate, wasted sludge) (Fig. 3F).

To evaluate the proliferation of ASVs when entering the digester, the shared ASVs per season were determined as well. Summer conditions showed 1146 shared ASVs (37.58%), among which ASV1 (classified as Ca *Brevefilum fermentans*) and ASV2 (classified as *Coprothermobacter proteolyticus*), were especially abundant (ASV1: 5.47 ± 6.49%, ASV2: 2.22 ± 4.15%) in the summer digestate. However, these ASVs only account for 0.21 ± 0.99% (ASV1) or 0.04 ± 0.21% (ASV2) of the summer WAS bacterial community. For winter conditions a different pattern emerges—only 651 ASVs (22.8%) are shared between the winter WAS and winter digestate samples, and, thus, remarkably fewer ASVs compared to summer counterparts. The shared ASV8, classified as MiDAS species 7162 (Rikenellaceae), was more abundantly present in the winter WAS samples (2.94 ± 2.68%) than the winter digestate (0.07 ± 0.05%).

The most abundant families represented in all samples were Comamonadaceae (average abundance across all samples: 5.26 ± 4.12%), Saprospiraceae (4.12 ± 4.13%), and Rhodocyclaceae (3.96 ± 4.54%). When zooming in on the digestate samples, Candidatus *Brevefilum* (4.34 ± 5.27%), *Coprothermobacter* (4.34 ± 10.55%) and the *Lentimicrobium* (5.81 ± 5.10%) genus were most abundant across all digestate samples. However, when looking at specific samples like wastewater treatment plant number 10 and 12, we can see a *Coprothermobacter* abundance of up to 48.09%. The most abundant genera in waste sludge samples were *MiDAS_g_6724* (2.44 ± 2.70%), *Rhodoferax* (2.11 ± 1.22%), and *Terrimonas* (2.10 ± 1.15%).

The bacterial primers used in this study were able to pick up the 16S rRNA genes of the most abundant members of methanogens as well [*in silico* the primers were shown to be able to cover all methanogens (110) present in the MiDAS database when allowing one mismatch in the forward primer (see supplementary information)], providing potentially a rudimentary insight into the methanogenic diversity present in the samples (Supplementary Fig. 1A). The most prominent genera across the different digestate samples were *Methanoculleus* and *Methanothrix* (formerly *Methanosaeta*). When looking at the total relative abundance of the methanogens in the samples, on average 1.00 ± 0.65% (maximum 2.81% and minimum 0.21%) of the microbial community (archaea and bacteria combined) were identified as methanogens. No significant difference between summer and winter methanogen abundances was detected (Wilcoxon rank sum test; *P* = .9097) (Supplementary Fig. 1B).

#### Alpha- and beta-diversity: the fingerprints

**Figure 4:**
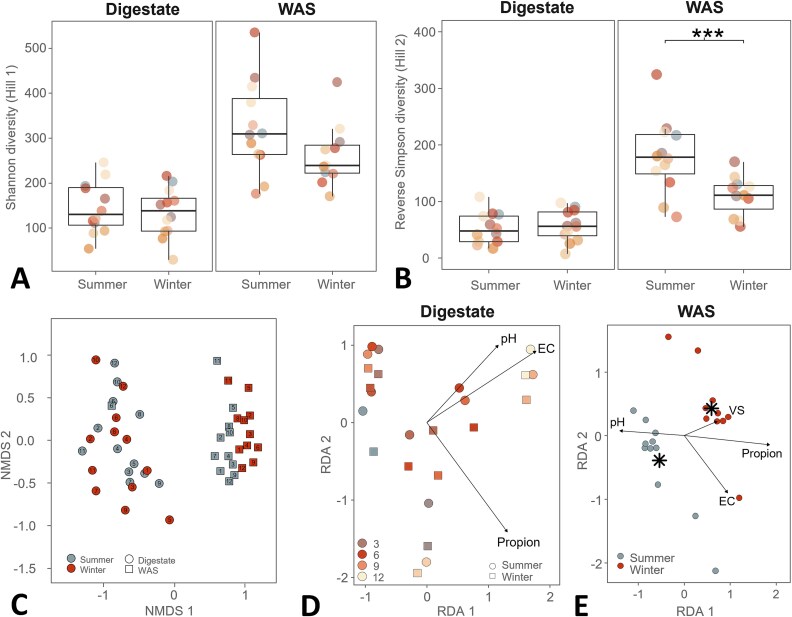
(A) Hill number D1 and (B) Hill number D2 of the bacterial communities compared over different sludge types and different seasons, significance was tested using a *t*-test (*P* < .05). (C) NMDS figure based on the Bray–Curtis statistic calculated using the bacterial genetic information of all samples. Numbers on the figure correspond to wastewater treatment plant number. Distance-based redundancy analysis (db-RDA) of the bacterial communities of (D) digestate samples and (E) waste activated sludge (WAS) samples using significant [calculated with (PERM)ANOVA (*P* < .05) (Table S7)] physicochemical parameters to explain the taxonomic diversity. In (D) the samples are colored based on the number of the wastewater treatment plants (from 1–12).

The alpha diversity of the taxonomic data showed that the WAS data was clearly more diverse both in terms of number of species (Fig. 4A) and evenness (Fig. 4B) than the digestate. When comparing over seasons, no significant difference was observed between the seasons for the digestate. Comparing WAS summer versus winter samples indicated that winter WAS samples showed significantly lower inverse Simpson values compared to their summer counterparts (*t*-test; *P* = .005926; Fig. 4B).

A clear grouping of the bacterial microbiome data could be observed when comparing the digestate and feed (WAS) samples (PERMANOVA: *P* = .001, perm: 999; Fig. 4C), however, a seasonal effect was less apparent, but significant nonetheless (PERMANOVA: *P* = .044, perm: 999). The bacterial community of the incoming WAS (feedstock of the digesters) showed grouping per season (PERMANOVA: *P* = .001, perm: 999) based on the Bray–Curtis dissimilarity matrix (beta-diversity; Fig. 4C). However, the microbiome of the digestate seems mainly grouped around sampling sites (WWTP number) (PERMANOVA: *P* = .006, perm: 999; Table S8), rather than based on the seasonality of the incoming sludge microbiome (PERMANOVA: *P* = .242, perm: 999; Fig. 4C) (Table S9). This grouping based on treatment plant is visible in the beta-diversity of the methanogenic taxonomy as well (Supplementary Fig. 1C). Three groups distinguish themselves based on treatment plant. This grouping is likely associated with the substrates used for methane production; groups 1 and 2 have more hydrogenotrophic members (high relative abundances of *Methanomassiliicoccus* and *Methanoculleus,* respectively), while group 3 has more acetoclastic and methylotrophic members (high relative abundances of *Methanotrix* and *Methanofastidium*, respectively).

A distance-based redundancy analysis (db-RDA) was performed to explain the (beta-)diversity using the physicochemical data across samples. For the WAS samples, the electrical conductivity, the pH, the propionic acid content, and the VS content significantly explained the taxonomic diversity, and demonstrated a clear clustering per season based on these parameters (Fig. 4E). For the digestate samples, the propionic acid content, electrical conductivity, and pH significantly influenced diversity, grouping was mainly based on the wastewater treatment plant itself (i.e. location, feed …), rather than the season (Fig. 4D).

#### Comparing conditions through composition and functionality

**Figure 5:**
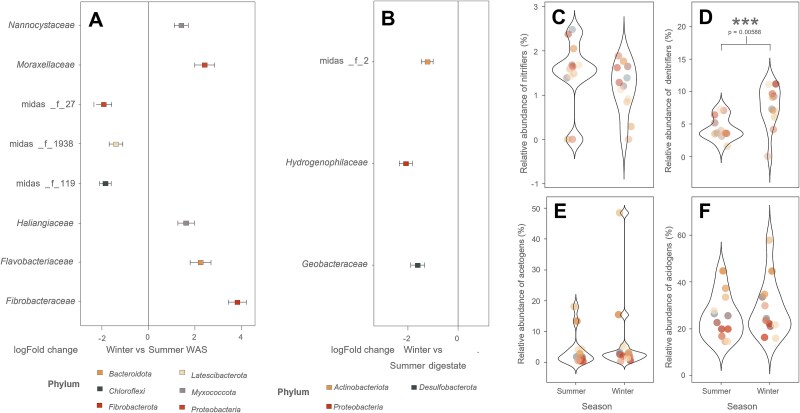
(A) and (B) show significantly different taxa when comparing the microbiomes of winter samples with summer samples for waste activated sludge (WAS) in (A) and digestate in (B) using the ANCOMB-C tool. Relative abundance of functional bacterial groups in the WAS samples compared over seasons colored by wastewater treatment plant, (C) nitrifiers (significant difference between seasons calculated using a paired Wilcoxon test, *P* = .2661) and (D) denitrifiers (significant difference between seasons calculated using a paired *t*-test, *P* = .04011). Relative abundance of functional bacterial groups in the digestate samples compared over seasons colored by waste water treatment plant (E) acetogens and (F) acidogens (fermenters).

Making use of the compositional comparison tool ANCOMB-C [35], digestate and WAS samples were separately compared across the two seasons to identify significantly different taxonomic families in both conditions (Fig. 5A and B). In the WAS samples, we see eight families differ significantly, representing 2.14 ± 1.35% of the total bacterial population in the summer samples and 9.01 ± 2.89% in the winter samples. Five of these families, *Haliangiaceae*, *Flavobacteriaceae*, *Fibrobacteraceae*, *Nannocystaceae* and *Moraxellaceae* had significantly more representatives present in the winter WAS samples than in the summer samples. Three other MiDAS families: 27, 119 and 1938, were more represented in the summer samples. These families belonged to *Proteobacteria, Chloroflexi*, and *Latescibacterota*, respectively. When comparing summer and winter digestate samples to each other, only three significantly different families were found. These families represent only 0.06 ± 0.06% of the total microbial composition of the winter digestate samples and only 0.39 ± 0.25% of the bacterial population of the summer samples. The three families, *Geobacteraceae*, *Hydrogenophilaceae*, and MiDAS family 2 (*Actinobacteriota*) were significantly more represented in the summer than in the winter samples.

In biological wastewater treatment, nitrification/denitrification is considered to be one of the most important functions of the bacterial biomass. To this end, the nitrifiers (Fig. 5C) and denitrifiers (Fig. 5D) were investigated in the WAS samples. Although there was no significant difference in the relative abundance of nitrifiers between summer and winter WAS samples, there was a significant (*P* = .012; Wilcoxon signed rank exact test) lower abundance of denitrifiers in the summer compared to winter (Fig. 5D).

The primary function of the digester is to break down organic material, not to carry out nitrification or denitrification. Therefore, the focus was placed on functional microbial groups involved in AD, including hydrolysers, acidogens, acetogens, and methanogens. No significant difference between winter and summer was apparent when comparing acidogens (fermenters) and acetogens over the seasons in the digestate samples (Wilcoxon signed rank exact test; *P* = .55 and *P* = .55; Fig. 5E and F).

## Discussion

In this study, an in-depth evaluation of the microbial community of 24 WAS streams (summer and winter) entering AD and 24 digestate samples from 12 full-scale AD installations were investigated to correlate microbial community composition and organization to the seasonal variation of the incoming stream. Specific differences in the physicochemical composition of both the incoming stream, as well as the digestate were observed. These physicochemical differences did not correlate to changes in the microbiome digestate however they did for the microbiome of the WAS.

### Seasonal variation influences waste activated sludge composition

Contrary to the popular saying “you are what you eat”, it seems that the microbiome of digesters fed WAS is less influenced by the bacterial composition of the incoming stream than generally assumed [37, 38]. We provide evidence here that WAS across different Belgian WWTPs is significantly affected by seasonality, which translates into significant changes in their physicochemical composition, i.e. pH, propionic acid and ammonium content, EC, and VS*.* This results in a shift during summer conditions toward a more diverse WAS bacterial community (inverse Simpson, more ASVs unique to summer samples). Certain families are more abundant at the start of winter compared to summer, such as *Flavobacteriaceae* and *Fibrobacteraceae,* as is the denitrifier community in general. However, this relative increase in putative denitrifiers could be explained by the fact that, functionally, denitrification is widespread across different bacteria clades [39]. Zhang *et al.* [19] showed that seasonality can have a significant effect on the functionality of wastewater treatment sludge (mainly on the nitrogen related functions) across different types of wastewater treatment facilities. Cao *et al.* [40] even observed sensitivity of the denitrification process to lower temperatures in the winter season, with a decrease in nitrogen removal of about 4% compared to summer. However, they also found that in some wastewater treatment facilities, nitrification was even more sensitive to a temperature drop over the seasons, resulting in a 2.5%–8.2% decrease in ammonium removal). The increase in diversity in summer observed in this study aligns with previous observations by Zhang *et al.* [19], suggesting that higher temperatures in open-air facilities can have a significant effect on the overall alpha diversity of the WWTP microbiome, at least in temperate climates.

### The digestate microbiome is less influenced by seasonality

WAS was used as a feedstock for all the digesters investigated within this study. A significant effect was observed on the inflowing WAS samples, due to the season, in terms of chemical composition and microbiome. The seasonal variation in the WAS had some effect on the chemistry of the digestate, (e.g. less acetate, more propionic acid, and an increase in total VFA content during winter). While this did result in grouping based on the physicochemical composition, it did not translate into a significant seasonal effect on the microbiome of the digesters. For example, the acetogenic methanogens *Methanosarcina* and *Methanothrix* did not differ significantly in relative archaeal abundance between summer and winter (21.08 ± 10.71% vs. 19.19 ± 12.10% for *Methanothrix* and 0.85 ± 2.95% vs. 0.98 ± 2.30% for *Methanosarcina*). The digester microbiome composition was more dependent on the plant it was sampled from. Compared across digesters, *Methanosarcina* was only present in two samples and a clear decrease from summer to winter of relative abundance of *Methanosarcina* seems present in WWTP 6 (from 10.22% to 6.04%). Similar grouping could be observed in the bacterial and the methanogenic beta-diversity plots (Fig. 4D and Supplementary Fig. 1). In methanogenic diversity plots, this grouping seemed related to the substrate use for methane production (hydrogenotrophic vs. methylotrophic/acetoclastic). This diversity across different facilities, while still having a similar functionality, was elsewhere observed by Mei *et al.* [38] and De Vrieze *et al.* [6], and has been discussed in reviews such as Vanwonterghem *et al.* [41]. One parameter of functionality that can be used to assess health and methane production in AD systems is the *Bacteroidetes* to *Firmicutes* ratio (B/F) B/F ratio did not increase over 4 for any of the digesters, suggesting that methanogenic functionality was not critically affected by seasonality. The percentage of detected methanogens in the total microbial population did not differ significantly between winter and summer samples. However, the average abundance of methanogens detected in the total microbial community was lower than would be expected in these systems and lower than reported in previous studies [42] (generally around 5% to 10% of the community in the digestate) [43]. This could be attributed to the bacterial primers used in the study [44], however, lower percentages of methanogens in full scale systems have been reported elsewhere [45]. Although such primers are not ideal for archaeal amplicon sequencing, these primers have been used to screen for archaea in other ecosystems, such as soil [44]. Wasimuddin *et al.*, [44] showed that allowing for a single mismatch rendered bacterial primers suitable for the detection of a representative number of archaea *in silico*, but that the community is underrepresented in the assignment of reads to archaea when testing in vivo.

### Common process parameters like volatile solids over total solids ratio and ammonium concentration did not alter the digestate microbiome or its performance

We observed a significant difference in VS/TS ratio in the WAS samples when comparing summer and winter conditions, which Wang *et al.* [15] had previously shown to have a significant effect on the microbial composition. In that study, an increase from a ratio of 0.35 to 0.56 was shown to alter the microbial activity, so that the functionality of the system itself showed an increasing methane production from 53.1 ± 3.3% to 57.6 ± 3.0%. Li *et al.* [46] found that when the VS proportion of feed sludge reached 60–65% at an organic loading rate of 3.50–3.70 g/(L·d), the AD exhibited the best performance with 32.19 ± 7.73% of the organic matter removed (influent-effluent/influent) and a methane production of 159 ± 13 mL/g added VS. They saw a clear difference in the methanogen composition toward a predominance of *Methanosarcina.* Nonetheless, in the present study, neither the *Bacteroidetes* to *Firmicutes* ratio, nor the total percentage of methanogens, nor the methanogenic composition alter significantly (DESEQ: padj >0.05, ANOSIM: *P* > .05, Supplementary Fig. 1) when the VS/TS increased in winter. This suggests that the number of methanogens and their functionality were not affected by this increase. Potentially, the increase in the efficiency of methane production (observed as an increase in activity) levels off above a certain VS/TS ratio. A similar trend was observed in a study by Wang *et al.* [15], when the observed methane production increase was greater when the VS/TS increased from 0.35 to 0.46 than when the VS/TS increased from 0.46 to 0.56.

We observed a significant increase in ammonium content comparing winter to summer digestate samples. Previous research [6, 46–48] strongly correlates an influence of ammonia concentration on methanogenic activity. Here, the average ammonium concentrations remained below 2 g.L^−1^ for most wastewater treatment facilities (> 2000 mg.L^−1^ TAN can already be toxic to unacclimated microbes), and it did not seem to have an effect on the microbial communities in the digestate (no significant effect based on db-RDA analysis, no clear grouping, Fig. 4). The seemingly non-significant effect of the ammonium concentration increase could be explained by an acclimatization of the microbial community. Lee *et al.* [46] found that *Methanoculleus* was dominant at high TAN concentrations, which is one of the most prominent methanogenic genera found in the sampled digesters in this study.

### Alpha diversity in feedstock does not translate into the digestate

Regardless of the alpha-diversity increase in summer in the WAS samples, this did not translate to an increase in alpha-diversity in the summer digestate. A grouping per season based on beta-diversity for the WAS also did not result in a grouping based on season in the digester, confirming the robustness of the digester communities sampled here. Despite the fluctuations in the inflow diversity and community, the digesters were able to maintain their diversity over the seasons. Contrasting, other studies found that the influent microbial community (the community of the feedstock) can be of significant influence on the structure of the microbial community in the digester [37, 46]. When comparing the two different sample types across seasons, parameters that affected the microbial communities of the feedstock (pH, ammonium, …) were significantly different in the digester samples over the two seasons as well, which did not translate into a proportionate influence on the digestate community. Across functional groups, there does not appear to be a significant difference between winter and summer. However, it is possible that the overall diversity across the twelve digester systems obscures changes in functionality. For separate digesters as a whole, there do appear to be seasonal differences which should be investigated further through more replicate sampling per digester across the different seasons.

## Supplementary Material

SupplementalData_forFinalSubmission_ycaf072

## Data Availability

The fastq files obtained from this study were submitted to the National Center for Biotechnology Information (NCBI) under the Accession number PRJNA1144789 (reviewer link: https://dataview.ncbi.nlm.nih.gov/object/PRJNA1144789?reviewer=remo96iv4uol1159b0vt79st1r). All other raw data (like chemical measurements) obtained during the study has been included in the supplemental Tables S1–S4.
