# Supplementary material for: The anaerobic digestion microbiome is robust toward variation in the waste activated sludge feed
Source: ISME Commun. 2025 Apr 25;6(1):ycaf072. doi: 10.1093/ismeco/ycaf072 (PMC13245180; doi:10.1093/ismeco/ycaf072)
Supplement: SupplementalData_forFinalSubmission_ycaf072 [file supplementaldata_forfinalsubmission_ycaf072.pdf]

The anaerobic digestion microbiome is robust towards variation in the waste activated sludge feed

Josefien Van Landuyt[1], Jasmine Oosterlinck[1,2], and Jo De Vrieze[1,3*]

[*] Corresponding author: Jo De Vrieze, Ghent University; Faculty of Bioscience Engineering; Centre for Microbial Ecology and Technology (CMET); Frieda Saeysstraat 1, B-9052 Gent, Belgium; phone: +32 (0)9 264 59 76; E-mail: Jo.DeVrieze@UGent.be; Webpage: www.cmet.ugent.be.

[1] Centre for Microbial Ecology and Technology (CMET), Universiteit Gent, Frieda Saeysstraat 1, B-9052 Gent, Belgium

[2] Witteveen+Bos Belgium N.V., Posthoflei 5, bus 1, 2600 Antwerpen – Berchem, Belgium

[3] Centre for Advanced Process Technology for Urban Resource Recovery (CAPTURE), Frieda Saeysstraat 1, B-9052 Gent, Belgium

Supplementary information

Table S1 – Listing of the included genera for the different functional groups for analysis between seasons

**Nitrifiers***	**Denitrifiers[1,2]****	**Acidogens***	**Acetogens***
Nitrosomonas	*Achromobacter*	*Acelobacterium*	*Acetitomaculum*
Nitrosococcus	*Actinobacillus*[3]	*Acetitomaculum*	*Acetoanaerobium*
Nitrosospira	*Actinomyces*[4]	*Acetivibrio*	*Acetonema*
Nitrobacter	*Aeromonas*[5]	*Acetoanaerobium*	*Anaerolinea*
Nitrospina [6]	*Agrobacterium*[7]	*Acetobacterium*	*Bellinea*
Nitrococcus [8]	*Alcaligenes*[9]	*Acetomicrobium*	*Blautia*
Nitrospira	*Arachnia*	*Acetonema*	*Ca Omnitrophus*
Nitrososphaera [10]	*Arthrobacter*	*Acholeplasma*	*Ca Phosphitivorax*
Nitrosopumilus [10]	*Azoarcus*[7]	*Acidaminobacter*	*Coprothermobacter*
Cenarchaeum [10]	*Azospirillum*[9]	*Acidaminococcus*	*Dehalobacterium*
Nitrosospongia [11]	*Bacillus*[9]	*Acidipropionibacterium*	*Levilinea*
Nitrosotalea[10]	*Bacterionema*	*Aciditerrimonas*	*Longilinea*
Nitrosotenuis[12]	*Bacteroides*[7]	*Actinomyces*	*Moorella*
Nitrosopelagicus [13]	*Beneckea*	*Aeromonas*	*Pelatomaculum*
Nitrosarchaeum[14]	*Bordetella*	*Agathobacter*	*Romboutsia*
Ca. Nitrohelix [15]	*Bradyrhizobium**	*Akkermansia*	*Ruminococcus*
Ca. Nitromaritima [16]	*Branhamella*	*Alcaligenes*	*Smithella*
Ca. Nitronauta [15]	*Brucella*[7]	*Alistipes*	*Sporosarcina*
Nitrolancetus [17]	*Burkholderia*[9]	*Eubacterium brachy group*	
Nitrotoga [18]	*Campylobacter*	*Alterococcus*	*Synthrophomonas*
	Cellulomonas	*Aminiphilus*	*Synthrophorhabdus*
	Chelatococcus	*Aminobacterium*	*Synthrophus*
	Chromobacterium[7]	*Aminomonas*	*Thermomarinilinea*
	Citrobacter	*Amphiplicatus*	
	Clostridium	*Anaerobranca*	
	Corynebacterium[7]	*Anaerocella*	
	Curvibacter	*Anaerofustis*	
	Cytophaga	*Anaerolinea*	
	Dactylsporangium	*Anaerosinus*	
	Dechloromonas[7]	*Anaerostipes*	
	Ensifer	*Anaerovibrio*	
	Enterobacter	*Anaerovorax*	
	Erwinia	*Arcobacter*	
	Escherichia	*Armatimonas*	
	Eubacterium	*Atopobium*	
	Flavobacterium[7]	*Azospirillum*	
	Fusobacterium	*Bacteroides*	
	Gemella	*Bavariicoccus*	
	Geodermatophilus	*Bellilinea*	
	Haemophilus	*Blautia*	
	Halobacterium[7]	*Blvii28 wastewater-sludge group*	

	Halococcus	*Bradyrhizobium*	
	Hyphomicrobium[7]	*Brochothrix*	
	Hyphomonas	*Brooklawnia*	
	Ideonella	*Butyricicoccus*	
	Klebsiella	*Butyrivibrio*	
	Lactobacillus	*Ca Amarolinea*	
	Leptothrix	*Ca Bipolaricaulis*	
	Listeria	*Ca Brevefilum*	
	Lucibacterium	*Ca Caldatribacterium*	
	Marinobacter[9]	*Ca Cloacimonas*	
	Marmoricola	*Ca Competibacter*	
	Methylobacterium	*Ca Fermentibacter*	
	Microbacterium	*Ca Obscuribacter*	
	Microbispora	*Ca Promineofilum*	
	Micrococcus	*Ca Saccharimonas*	
	Micromonospora	*Caldicoprobacter*	
	Moraxella[7]	*Caldilinea*	
	Mycobacterium	*Caloramator*	
	Neisseria[7]	*Capnocytophaga*	
	Nisaea	*Carnobacterium*	
	Nocardia	*Catenibacterium*	
	Paracoccus[9]	*Cloacibacillus*	
	Pasteurella	*Cloacibacterium*	
	Peptococcus	*Cloacimonas*	
	Photobacterium	*Clostridioides*	
	Planobispora	*Clostridium*	
	Planomonospora	*Clostridium sensu stricto 1*	
	Plesiomonas	*Colidextribacter*	
	Propionibacterium	*Coprococcus*	
	Proteus	*Coprothermobacter*	
	Pseudogulbenkiania	*Corynebacterium*	
	Pseudomonas[9]	*Crenothrix*	
	Pusillimonas	*Cutibacterium*	
	Ralstonia[9]	*Defluviicoccus*	
	Rhizobium[9]	*Defluviimonas*	
	Rhodobacter[9]	*Fimbrimonas*	
	Rothia	*Defluviitalea*	
	Rubrivivax[9]	*Faecalibacterium*	
	Salmonella	*Defluviitoga*	
	Selenomonas	*Desulfobulbus*	
	Serratia	*Desulfococcus*	
	Shewanella[9]	*Eubacterium nodatum group*	
	Shigella	*Desulfomicrobium*	
	Simonsiella	*Desulforhopalus*	
	Sinorhizobium[7]	*Desulfotomaculum*	
	Spirillum[7]	*Desulfovibrio*	

	Sporosarcina	*Dorea*	
	Staphylococcus	*Dyadobacter*	
	Streptomyces[9]	*Dysgonomonas*	
	Streptosporangium	*Enhydrobacter*	
	Thauera[9]	*Fibrobacter*	
	Thiobacillus[7]	*Enterococcus*	
	Thiomicrospira[7]	*Ercella*	
	Veillonella	*Erysipelatoclostridium*	
	Vibrio	*Erysipelothrix*	
	Zoogloea	*Eubacterium*	
		Flavobacterium	
		Flexilinea	
		Fusibacter	
		Fusicatenibacter	
		Gelria	
		Gemmobacter	
		Geobacter	
		Geothrix	
		Gimesia	
		Gracilibacter	
		Granulicatella	
		Hahella	
		Halomonas	
		Herbinix	
		Herpetosiphon	
		Holdemanella	
		Hungateiclostridium	
		Hyphomicrobium	
		Hypnocyclicus	
		Ignavibacterium	
		Intestinibacter	
		Kouleothrix	
		Lachnoclostridium	
		Lachnospira	
		Lachnospiraceae AC2044 group	
		Lachnospiraceae NK3A20 group	
		Lachnospiraceae NK4A136 group	
		Lachnospiraceae UCG-004	
		Lacticaseibacillus	
		Lactiplantibacillus	
		Lactivibrio	
		Lactobacillus	
		Lactococcus	
		Lactovibrio	
		Lautropia	
		Lentimicrobium	

		Leptolinea	
		Leptotrichia	
		Leuconostoc	
		Levilinea	
		Ligilactobacillus	
		Limnochorda	
		Limosilactobacillus	
		Litorilinea	
		Longilinea	
		Longivirga	
		Lutaonella	
		Luteococcus	
		Lutispora	
		Macelibacteroides	
		Mariniphaga	
		Megamonas	
		Megasphaera	
		Mesotoga	
		Mesotoga	
		Micropruina	
		Miniimonas	
		Mitsuokella	
		Mobilitalea	
		Monoglobus	
		Moorella	
		Mycobacterium	
		Natronoflexus	
		Nocardioides	
		OLB13	
		OLB8	
		Omatilinea	
		Opitutus	
		Oscillibacter	
		Ottowia	
		Paeniclostridium	
		Paludibacter	
		Paludibacterium	
		Pannonibacter	
		Papillibacter	
		Parabacteroides	
		Parabukholderia	
		Paraclostridium	
		Pelolinea	
		Pelotomaculum	
		Peptoclostridium	
		Peptostreptococcus	

		Petrimonas	
		Phaeodactylibacter	
		Phascolarctobacterium	
		Planifilum	
		Pontibacter	
		Prevotella	
		Propionibacterium	
		Propionicicella	
		Propioniciclava	
		Propionicimonas	
		Propionispira	
		Propionivibrio	
		Proteiniborus	
		Proteiniclasticum	
		Proteiniphilum	
		Proteinivorax	
		Proteocatella	
		Pseudobacteroides	
		Raineyella	
		Rhizorhapis	
		Rhodoferax	
		Rhodopseudomonas	
		Romboutsia	
		Roseburia	
		Roseomonas	
		Rothia	
		Rubinisphaera	
		Rubrivivax	
		Ruminoclostridium	
		Ruminococcus	
		Ruminococcus gauvreauii group	
		Ruminococcus gnavus group	
		Ruminococcus torques group	
		Runella	
		Saccharicrinis	
		Saccharofermentans	
		Salinispira	
		Schleiferilactobacillus	
		Sebaldella	
		Sedimentibacter	
		Selenomonas	
		Sellimonas	
		Serratia	
		Shuttleworthia	
		Solobacterium	
		Sorangium	

		Sphaerotilus	
		Spirochaeta	
		Sporanaerobacter	
		Staphylococcus	
		Streptococcus	
		Subdoligranulum	
		Sulfurospirillum	
		Sumerlaea	
		Sunxiuqinia	
		Syntrophobacter	
		Syntrophus	
		Tangfeifania	
		Tepidimicrobium	
		Terrisporobacter	
		Tessaracoccus	
		Tetrasphaera	
		Thermanaerovibrio	
		Thermoactinomyces	
		Thermoanaerobaculum	
		Thermogutta	
		Thermomonas	
		Thermosipho	
		Thermovirga	
		Tissierella	
		TM7x	
		Tolumonas	
		Treponema	
		Trichococcus	
		Truepera	
		Turicibacter	
		Tyzzerella	
		UBA1819	
		Uruburuella	
		Vagococcus	
		Veillonella	
		Weissella	
		XBB1006	
		Zoogloea	

*nitrifier, acidogens/fermenters, and acetogens list based on MIDAS field guide database (https://www.midasfieldguide.org/guide/search) of genera capable of the selected function but adjusted and genera added based on the mentioned references.

**denitrifier list was based on the list of Tiedje (1988) but adjusted and genera added based on the mentioned references

Table S2 - Chemical parameters waste activated sludge winter samples (n = 3).

SUMMER Feedstock	TS (g/L)	VS (g/L)	Total COD (gO_2/L)	VS/TS (-)	COD/VS (-)
**Average**	61.7 ± 1.7	35.9 ± 1.0	73.2 ± 4.6	0.58 ± 0.00	2.02 ± 0.21
**RWZI 1**	78.4 ± 1.5	43.9 ± 1.0	79.3 ± 7.7	0.56 ± 0.00	1.81 ± 0.23
**RWZI 2**	68.5 ± 0.1	40.2 ± 0.2	86.0 ± 7.9	0.59 ± 0.00	2.14 ± 0.25
**RWZI 3**	63.7 ± 0.0	42.9 ± 0.1	104.3 ± 8.4	0.67 ± 0.00	2.43 ± 0.25
**RWZI 4**	76.6 ± 0.2	43.0 ± 0.0	99.3 ± 3.1	0.56 ± 0.00	2.31 ± 0.09
**RWZI 5**	68.7 ± 3.4	41.8 ± 2.0	82.3 ± 8.7	0.61 ± 0.00	1.97 ± 0.30
**RWZI 6**	49.3 ± 1.8	27.2 ± 1.0	58.2 ± 0.7	0.55 ± 0.00	2.14 ± 0.10
**RWZI 7**	58.8 ± 6.2	35.1 ± 3.8	88.4 ± 0.3	0.60 ± 0.00	2.52 ± 0.35
**RWZI 8**	54.7 ± 1.9	32.8 ± 1.3	77.8 ± 3.0	0.60 ± 0.00	2.37 ± 0.17
**RWZI 9**	50.5 ± 4.0	29.4 ± 2.3	45.0 ± 2.5	0.58 ± 0.00	1.53 ± 0.19
**RWZI 10**	48.1 ± 0.1	26.8 ± 0.1	38.1 ± 4.0	0.56 ± 0.00	1.42 ± 0.20
**RWZI 11**	57.2 ± 0.4	37.6 ± 0.4	71.0 ± 0.8	0.66 ± 0.00	1.89 ± 0.04
**RWZI 12**	65.3 ± 0.8	29.7 ± 0.5	48.9 ± 8.1	0.45 ± 0.00	1.65 ± 0.36

SUMMER Feedstock	pH (-)	EC (mS/cm)	Total VFA (mgCOD/L)	Acetic acid (%totaal)	Propionic acid (%totaal)	$[NH_4^+]$ (gNH_4^+-N/L)
**Average**	7.03 ± 0.01	4.45 ± 0.05	1219 ± 137	79 ± 3 %	6 ± 0 %	0.65 ± 0.01
**RWZI 1**	6.87 ± 0.00	3.24 ± 0.02	73 ± 24	79 ± 21 %	0 ± 0 %	0.44 ± 0.03
**RWZI 2**	6.94 ± 0.00	3.79 ± 0.03	98 ± 12	94 ± 6 %	0 ± 0 %	0.55 ± 0.01
**RWZI 3**	6.91 ± 0.01	2.92 ± 0.04	45 ± 1	100 ± 0 %	0 ± 0 %	0.44 ± 0.01
**RWZI 4**	6.92 ± 0.01	3.11 ± 0.02	50 ± 2	100 ± 0 %	0 ± 0 %	0.46 ± 0.01
**RWZI 5**	6.92 ± 0.01	4.82 ± 0.01	4512 ± 303	39 ± 1 %	31 ± 0 %	0.84 ± 0.00
**RWZI 6**	7.70 ± 0.05	9.98 ± 0.15	387 ± 10	77 ± 0 %	14 ± 0 %	1.62 ± 0.00
**RWZI 7**	7.11 ± 0.01	5.07 ± 0.02	62 ± 2	100 ± 0 %	0 ± 0 %	0.87 ± 0.00
**RWZI 8**	6.94 ± 0.00	2.16 ± 0.05	56 ± 4	100 ± 0 %	0 ± 0 %	0.25 ± 0.01
**RWZI 9**	7.14 ± 0.01	4.46 ± 0.13	75 ± 6	52 ± 2 %	0 ± 0 %	0.56 ± 0.00
**RWZI 10**	7.10 ± 0.00	4.38 ± 0.13	57 ± 9	100 ± 0 %	0 ± 0 %	0.58 ± 0.00
**RWZI 11**	6.84 ± 0.01	7.08 ± 0.00	9179 ± 1268	2 ± 0 %	29 ± 1 %	1.03 ± 0.00
**RWZI 12**	6.98 ± 0.02	2.36 ± 0.02	34 ± 0	100 ± 0 %	0 ± 0%	0.19 ± 0.00

Table S3 – Chemical parameters waste activated sludge winter samples (n = 3).

WINTER Feedstock	TS (g/L)	VS (g/L)	Total COD (gO_2/L)	VS/TS (-)	COD/VS (-)
Average	63.5 ± 1.4	42.0 ± 1.0	58.8 ± 3.6	0.65 ± 0.00	1.46 ± 0.20
RWZI 1	73.2 ± 0.5	43.7 ± 0.3	48.2 ± 3.8	0.60 ± 0.00	1.10 ± 0.51
RWZI 2	68.0 ± 0.5	45.2 ± 0.2	58.9 ± 1.9	0.66 ± 0.00	1.30 ± 0.08
RWZI 3	62.7 ± 0.5	46.3 ± 0.4	63.2 ± 1.7	0.74 ± 0.00	1.37 ± 0.07
RWZI 4	58.4 ± 2.4	38.6 ± 1.7	82.9 ± 2.8	0.66 ± 0.00	2.15 ± 0.21
RWZI 5	64.2 ± 0.2	42.9 ± 1.5	45.8 ± 16.1	0.67 ± 0.02	1.07 ± 0.65
RWZI 6	56.2 ± 0.6	40.6 ± 0.6	43.2 ± 2.5	0.72 ± 0.00	1.06 ± 0.11
RWZI 7	80.0 ± 0.0	58.0 ± 0.0	54.9 ± 2.8	0.73 ± 0.00	0.95 ± 0.08
RWZI 8	81.6 ± 0.7	49.3 ± 0.5	53.9 ± 3.4	0.60 ± 0.00	1.09 ± 0.12
RWZI 9	61.9 ± 1.6	41.6 ± 1.1	62.3 ± 1.6	0.67 ± 0.00	1.50 ± 0.10
RWZI 10	50.6 ± 0.1	34.1 ± 0.2	58.2 ± 1.8	0.53 ± 0.01	1.71 ± 0.09
RWZI 11	44.8 ± 4.4	32.1 ± 3.0	71.2 ± 2.0	0.72 ± 0.00	2.22 ± 0.38
RWZI 12	60.2 ± 4.8	32.1 ± 2.6	62.5 ± 2.9	0.53 ± 0.00	1.95 ± 0.32

WINTER Feedstock	pH (-)	EC (mS/cm)	Total VFA (mgCOD/L)	Acetic acid (%totaal)	Propionic acid (%totaal)	$[NH_4^+]$ (gNH_4^+-N/L)
Average	6.46 ± 0.01	4.03 ± 0.09	4855 ± 187	41 ± 0 %	29 ± 0 %	0.74 ± 0.01
RWZI 1	6.99 ± 0.02	4.44 ± 0.07	3905 ± 362	28 ± 1 %	26 ± 0 %	1.54 ± 0.02
RWZI 2	6.32 ± 0.00	3.42 ± 0.08	3487 ± 133	35 ± 1 %	29 ± 0 %	0.58 ± 0.01
RWZI 3	6.13 ± 0.01	3.22 ± 0.02	5242 ± 76	37 ± 0 %	26 ± 0 %	0.67 ± 0.00
RWZI 4	7.01 ± 0.01	3.64 ± 0.08	4514 ± 60	29 ± 1%	35 ± 0 %	0.63 ± 0.00
RWZI 5	6.75 ± 0.01	4.02 ± 0.17	6603 ± 358	43 ± 1 %	31 ± 0 %	0.75 ± 0.00

**RWZI 6**	6.33 ± 0.03	3.20 ± 0.05	5551 ± 246	42 ± 0 %	31 ± 0 %	0.53 ± 0.08
**RWZI 7**	6.57 ± 0.04	3.60 ± 0.07	3507 ± 259	27 ± 1 %	37 ± 0 %	0.68 ± 0.00
**RWZI 8**	7.00 ± 0.00	3.18 ± 0.08	2442 ± 123	24 ± 0 %	46 ± 0%	0.55 ± 0.00
**RWZI 9**	5.81 ± 0.02	5.13 ± 0.19	7547 ± 127	43 ± 0 %	28 ± 0 %	0.72 ± 0.01
**RWZI 10**	6.05 ± 0.01	5.23 ± 0.10	7995 ± 371	47 ± 0 %	31 ± 0 %	0.82 ± 0.01
**RWZI 11**	5.67 ± 0.01	5.54 ± 0.09	7426 ± 120	39 ± 0 %	26 ± 0 %	0.85 ± 0.00
**RWZI 12**	6.94 ± 0.01	3.79 ± 0.13	42 ± 4	100 ± 0 %	0 ± 0 %	0.58 ± 0.00

Table S4 – Chemical parameters digestate summer samples (n = 3).

**SUMMER Digestate**	**TS (g/L)**	**VS (g/L)**	**VS/TS (-)**	**pH (-)**	**EC (mS/cm)**
**Average**	51.3 ± 1.8	26.8 ± 0.9	0.53 ± 0.00	7.85 ± 0.01	9.18 ± 0.10
**RWZI 1**	52.8 ± 2.2	28.1 ± 1.1	0.53 ± 0.00	7.54 ± 0.00	7.25 ± 0.04
**RWZI 2**	57.5 ± 0.1	31.3 ± 0.0	0.54 ± 0.00	7.63 ± 0.01	8.38 ± 0.15
**RWZI 3**	45.5 ± 0.1	27.7 ± 0.1	0.61 ± 0.00	7.77 ± 0.00	9.46 ± 0.05
**RWZI 4**	59.9 ± 0.1	30.9 ± 0.1	0.52 ± 0.00	7.79 ± 0.00	8.29 ± 0.06
**RWZI 5**	38.6 ± 2.4	19.6 ± 1.3	0.51 ± 0.00	7.78 ± 0.01	8.40 ± 0.03
**RWZI 6**	49.5 ± 4.1	27.6 ± 2.4	0.56 ± 0.00	8.06 ± 0.03	9.96 ± 0.10
**RWZI 7**	49.8 ± 3.9	26.0 ± 2.0	0.52 ± 0.00	7.87 ± 0.00	8.72 ± 0.08
**RWZI 8**	59.1 ± 3.9	32.2 ± 2.1	0.54 ± 0.00	7.66 ± 0.00	8.31 ± 0.02
**RWZI 9**	45.3 ± 1.4	24.2 ± 0.7	0.54 ± 0.00	8.01 ± 0.01	8.75 ± 0.24
**RWZI 10**	55.4 ± 3.3	25.8 ± 0.2	0.47 ± 0.00	8.02 ± 0.01	11.25 ± 0.08
**RWZI 11**	41.5 ± 0.3	24.9 ± 0.3	0.60 ± 0.00	7.87 ± 0.01	9.03 ± 0.14
**RWZI 12**	60.4 ± 0.3	23.1 ± 0.4	0.38 ± 0.00	8.22 ± 0.01	12.34 ± 0.18

**SUMMER** Digestate	Total VFA (mgCOD/L)	Acetic acid (%)	Propionic acid (%)	$[NH_4^+]$ (gNH_4^+-N/L)
**Average**	146 ± 12	80 ± 4 %	8 ± 2 %	1.45 ± 0.02
**RWZI 1**	101 ± 3	100 ± 0 %	0 ± 0 %	1.27 ± 0.08
**RWZI 2**	184 ± 24	73 ± 3 %	13 ± 2 %	1.41 ± 0.05
**RWZI 3**	127 ± 4	74 ± 6 %	0 ± 0 %	1.70 ± 0.01
**RWZI 4**	231 ± 5	75 ± 1 %	14 ± 1 %	1.52 ± 0.01
**RWZI 5**	48 ± 5	100 ± 0 %	0 ± 0%	1.43 ± 0.04
**RWZI 6**	180 ± 6	78 ± 3 %	16 ± 0 %	1.61 ± 0.01
**RWZI 7**	69 ± 3	100 ± 0 %	0 ± 0 %	1.43 ± 0.01
**RWZI 8**	197 ± 6	88 ± 1 %	0 ± 0 %	1.48 ± 0.03
**RWZI 9**	136 ± 22	37 ± 1 %	0 ± 0%	1.18 ± 0.00
**RWZI 10**	111 ± 18	79 ± 1 %	21 ± 1%	1.50 ± 0.02
**RWZI 11**	334 ± 58	69 ± 12%	17 ± 2%	1.22 ± 0.00
**RWZI 12**	29 ± 6	85 ± 15%	15 ± 15%	1.64 ± 0.00

Table S5 – Chemical parameters digestate winter samples (n = 3).

**WINTER** Digestate	Total VFA (mgCOD/L)	Acetic acid (%)	Propionic acid (%)	$[NH_4^+]$ (gNH_4^+-N/L)
**Average**	162 ± 13	50 ± 5 %	14 ± 1 %	1.64 ± 0.04
**RWZI 1**	231 ± 6	44 ± 0 %	11 ± 0 %	1.07 ± 0.06
**RWZI 2**	2328 ± 6	39 ± 2 %	16 ± 1 %	1.66 ± 0.07
**RWZI 3**	170 ± 13	45 ± 3 %	14 ± 1 %	2.07 ± 0.07
**RWZI 4**	226 ± 2	42 ± 1 %	16 ± 0 %	2.15 ± 0.09
**RWZI 5**	57 ± 7	33 ± 17 %	0 ± 0 %	1.32 ± 0.01
**RWZI 6**	238 ± 10	48 ± 0 %	18 ± 0 %	1.68 ± 0.01
**RWZI 7**	122 ± 29	64 ± 8 %	21 ± 1 %	1.78 ± 0.01
**RWZI 8**	90 ± 13	69 ± 0 %	21 ± 0 %	1.85 ± 0.07
**RWZI 9**	82 ± 6	74 ± 4 %	0 ± 0 %	1.48 ± 0.01
**RWZI 10**	187 ± 6	61 ± 3 %	15 ± 0 %	1.73 ± 0.01

**RWZI 11**	59 ± 3	42 ± 1 %	28 ± 0 %	1.17 ± 0.01
**RWZI 12**	262 ± 62	37 ± 10 %	12 ± 3 %	1.76 ± 0.01

**WINTER** Digestate	TS (g/L)	VS (g/L)	VS/TS (-)	pH (-)	EC (mS/cm)
**Average**	47.5 ± 1.1	26.0 ± 0.7	0.56 ± 0.01	7.78 ± 0.02	9.17 ± 0.09
**RWZI 1**	53.3 ± 0.2	29.0 ± 0.1	0.54 ± 0.00	7.59 ± 0.03	7.40 ± 0.18
**RWZI 2**	46.0 ± 0.2	25.9 ± 0.2	0.56 ± 0.00	7.71 ± 0.01	7.83 ± 0.04
**RWZI 3**	43.3 ± 0.3	26.8 ± 0.1	0.61 ± 0.00	7.85 ± 0.01	8.41 ± 0.07
**RWZI 4**	56.9 ± 1.2	31.1 ± 0.8	0.55 ± 0.00	8.01 ± 0.01	11.49 ± 0.04
**RWZI 5**	46.3 ± 0.6	26.1 ± 0.5	0.56 ± 0.00	7.64 ± 0.02	7.74 ± 0.09
**RWZI 6**	43.0 ± 0.2	25.8 ± 0.1	0.60 ± 0.00	7.90 ± 0.01	9.14 ± 0.10
**RWZI 7**	47.2 ± 3.0	24.5 ± 2.6	0.52 ± 0.02	7.80 ± 0.02	9.69 ± 0.16
**RWZI 8**	50.8 ± 3.4	26.8 ± 1.8	0.53 ± 0.00	7.90 ± 0.02	8.97 ± 0.03
**RWZI 9**	46.1 ± 0.2	26.2 ± 0.2	0.57 ± 0.01	7.77 ± 0.04	9.94 ± 0.17
**RWZI 10**	44.5 ± 0.3	23.4 ± 0.4	0.67 ± 0.00	7.84 ± 0.03	10.61 ± 0.08
**RWZI 11**	32.7 ± 3.9	19.0 ± 1.3	0.59 ± 0.03	7.52 ± 0.00	7.72 ± 0.04
**RWZI 12**	59.9 ± 0.0	27.5 ± 0.1	0.46 ± 0.00	7.79 ± 0.03	11.13 ± 0.04

Table S6 – Excerpt of PCA testing on the chemical variables

```
Sampling bootstrap replicates... Please wait

Calculating confidence intervals of empirical statistics... Please wait

Sampling random permutations... Please wait

Comparing empirical statistics with their null distributions... Please wait

========================================================
Test of PCA significance: 9 variables, 48 observations
100 bootstrap replicates, 100 random permutations
========================================================

Empirical Psi = 19.9279, Max null Psi = 2.4162, Min null Psi = 0.6902, p-value = 0
Empirical Phi = 0.5261, Max null Phi = 0.1832, Min null Phi = 0.0979, p-value = 0

Empirical eigenvalue #1 = 4.83247, Max null eigenvalue = 2.16024, p-value = 0
Empirical eigenvalue #2 = 2.08396, Max null eigenvalue = 1.67325, p-value = 0
Empirical eigenvalue #3 = 0.9219, Max null eigenvalue = 1.44893, p-value = 1
Empirical eigenvalue #4 = 0.4822, Max null eigenvalue = 1.32359, p-value = 1
Empirical eigenvalue #5 = 0.29835, Max null eigenvalue = 1.12762, p-value = 1
Empirical eigenvalue #6 = 0.22484, Max null eigenvalue = 0.98903, p-value = 1
Empirical eigenvalue #7 = 0.08083, Max null eigenvalue = 0.84906, p-value = 1
Empirical eigenvalue #8 = 0.0585, Max null eigenvalue = 0.70761, p-value = 1
Empirical eigenvalue #9 = 0.01694, Max null eigenvalue = 0.61724, p-value = 1
```

PC 1 is significant and accounts for 53.7% (95%-CI:46.1-61.9) of the total variation
PC 2 is significant and accounts for 23.2% (95%-CI:16.6-31) of the total variation

The first 2 PC axes are significant and account for 76.8% of the total variation

Variables 1, 2, 3, 4, 5, 6, 7, 8, and 9 have significant loadings on PC 1
Variables 7, and 8 have significant loadings on PC 2

Table S7 – ANOVA showing the significance of the different environmental parameters added to the distance-based RDA model.

```
Permutation test for dbrda under reduced model
Terms added sequentially (first to last)
Permutation: free
Number of permutations: 999

Model: vegan::dbrda(formula = rdaplot ~ TS + VS + pH + EC + Propion +
WWTP + TotalVFA_SE, data = sampleinfo, distance = "bray")
```

	Df	SumOfSqs	F	Pr(>F)	
**TS**	1	1,8364	7,6	0	***
**VS**	1	1,6222	6,714	0	***
**pH**	1	1,0752	4,45	0	***
**EC**	1	1,1137	4,609	0	***
**Propion**	1	0,5614	2,323	0,01	*
**WWTP**	1	0,5783	2,394	0,01	**
**TotalVFA_SE**	1	0,3978	1,647	0,03	*
Residual	39	9,4235			

```
---
Signif. codes:  0 '***' 0.001 '**' 0.01 '*' 0.05 '.' 0.1 ' ' 1
```

Table S8 – PERMANOVA showing the significance of the WWTP facility for the digestate

```
Permutation: free
Number of permutations: 999

Terms added sequentially (first to last)

          Df SumsOfSqs MeanSqs F.Model      R2 Pr(>F)
WWTP       1    0.7453 0.74533   2.738 0.11068  0.006 **
Residuals 22    5.9888 0.27222         0.88932
Total     23    6.7341                 1.00000
---
Signif. codes:  0 '***' 0.001 '**' 0.01 '*' 0.05 '.' 0.1 ' ' 1
```

Table S9 – Bray-curtis dissimilarity matrix comparing averaging the samples per condition (sludge and season)

	Digestate_Summer	Digestate_Winter	WAS_Summer
Digestate_Winter	0.3272447		
WAS_Summer	0.7657948	0.8130261	
WAS_Winter	0.8740921	0.8803241	0.5114266

1. Tugtas, A. E. Effect of Nitrate Reduction on the Methanogenic Fermentation: Process Interactions and Modeling. (2007).

2. Tiedje, J. Ecology of denitrification and dissimilatory nitrate reduction to ammonium. in *Methods of Soil Analysis. Part 2. Chemical and Microbiological Properties* vol. 717 179–244 (1988).
3. Jones, C. M. Denitrification: from genes to ecosystems. *Acta Univ. Agric. Sueciae* (2010).
4. Zhang, M., He, T., Wu, P., Wang, C. & Zheng, C. Recent advances in the nitrogen cycle involving actinomycetes: Current situation, prospect and challenge. *Bioresour. Technol.* **419**, 132100 (2025).
5. Ngara, T. R., Zeng, P. & Zhang, H. Biological Nitrogen Removal Database: A Manually Curated Data Resource. *Microorganisms* **10**, 431 (2022).
6. Lücker, S. & Daims, H. The Family Nitrospinaceae. in *The Prokaryotes: Deltaproteobacteria and Epsilonproteobacteria* (eds. Rosenberg, E., DeLong, E. F., Lory, S., Stackebrandt, E. & Thompson, F.) 231–237 (Springer, Berlin, Heidelberg, 2014). doi:10.1007/978-3-642-39044-9_402.
7. Philippot, L., Hallin, S. & Schloter, M. Ecology of Denitrifying Prokaryotes in Agricultural Soil. in *Advances in Agronomy* vol. 96 249–305 (Academic Press, 2007).
8. Prosser, J. I. NITROGEN IN SOILS | Nitrification. in *Encyclopedia of Soils in the Environment* (ed. Hillel, D.) 31–39 (Elsevier, Oxford, 2005). doi:10.1016/B0-12-348530-4/00512-9.
9. Mrkonjic Fuka, M., Gesche Braker, S. H. & Philippot, L. Chapter 20 - Molecular Tools to Assess the Diversity and Density of Denitrifying Bacteria in Their Habitats. in *Biology of the Nitrogen Cycle* (eds. Bothe, H., Ferguson, S. J. & Newton, W. E.) 313–330 (Elsevier, Amsterdam, 2007). doi:10.1016/B978-044452857-5.50021-7.
10. Hatzenpichler, R. Diversity, Physiology, and Niche Differentiation of Ammonia-Oxidizing Archaea. *Appl. Environ. Microbiol.* **78**, 7501–7510 (2012).

11. Moeller, F. U. *et al.* Characterization of a thaumarchaeal symbiont that drives incomplete nitrification in the tropical sponge Ianthella basta. *Environ. Microbiol.* **21**, 3831–3854 (2019).

12. Lebedeva, E. V. *et al.* Enrichment and genome sequence of the group I.1a ammonia-oxidizing Archaeon 'Ca. Nitrosotenuis uzonensis' representing a clade globally distributed in thermal habitats. *PloS One* **8**, e80835 (2013).

13. Santoro, A. E., Bayer, B., Elling, F. J. & Pearson, A. Candidatus Nitrosopelagicus. in *Bergey's Manual of Systematics of Archaea and Bacteria* 1–13 (John Wiley & Sons, Ltd, 2021). doi:10.1002/9781118960608.gbm01969.

14. Jung, M.-Y., Islam, M. A., Gwak, J.-H., Kim, J.-G. & Rhee, S.-K. Nitrosarchaeum koreense gen. nov., sp. nov., an aerobic and mesophilic, ammonia-oxidizing archaeon member of the phylum Thaumarchaeota isolated from agricultural soil. *Int. J. Syst. Evol. Microbiol.* **68**, 3084–3095 (2018).

15. Mueller, A. J. *et al.* Genomic and kinetic analysis of novel Nitrospinae enriched by cell sorting. *ISME J.* **15**, 732–745 (2021).

16. Ngugi, D. K., Blom, J., Stepanauskas, R. & Stingl, U. Diversification and niche adaptations of Nitrospina-like bacteria in the polyextreme interfaces of Red Sea brines. *ISME J.* **10**, 1383–1399 (2016).

17. Sorokin, D. Y. *et al.* Nitrification expanded: discovery, physiology and genomics of a nitrite-oxidizing bacterium from the phylum Chloroflexi. *ISME J.* **6**, 2245–2256 (2012).

18. Elling, F. J. *et al.* Marine and terrestrial nitrifying bacteria are sources of diverse bacteriohopanepolyols. *Geobiology* **20**, 399–420 (2022).

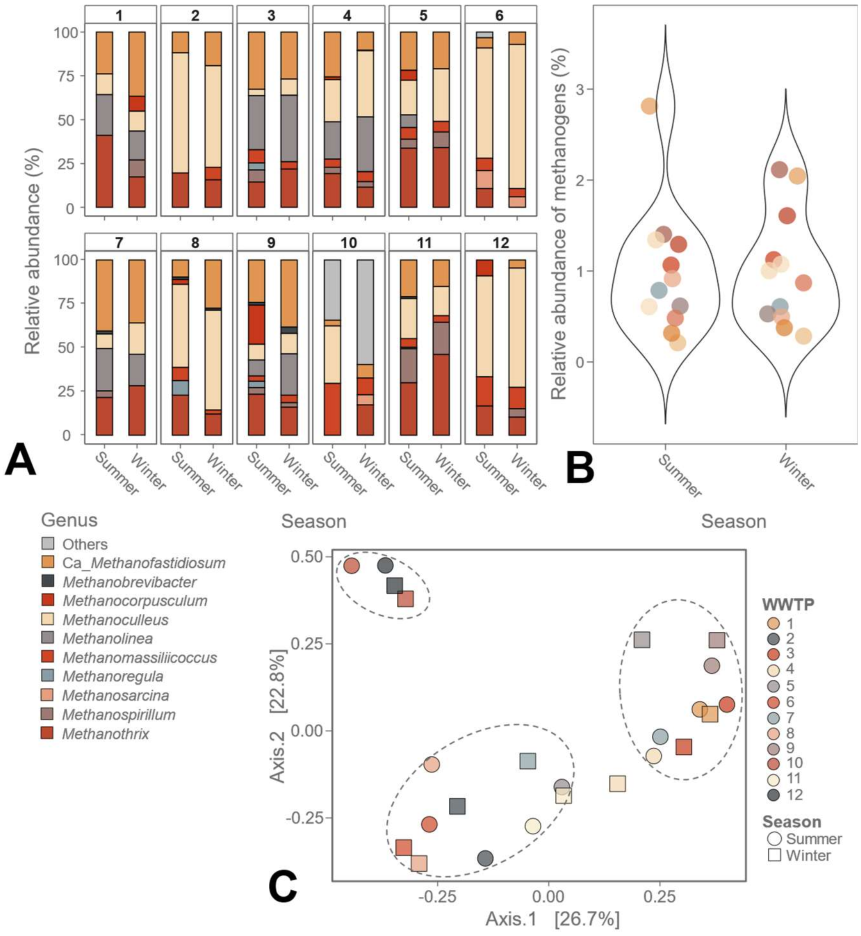

Supplementary Figure 1 – **A**. Relative abundance of top ten methanogenic genera per waste water treatment plant (WWTP) and per season. **B**. Relative abundance of the methanogens in the total microbial community of the digestate samples compared over seasons coloured by WWTP. **C**. PCoA figure of the beta-diversity of the methanogenic composition of the digestate samples.

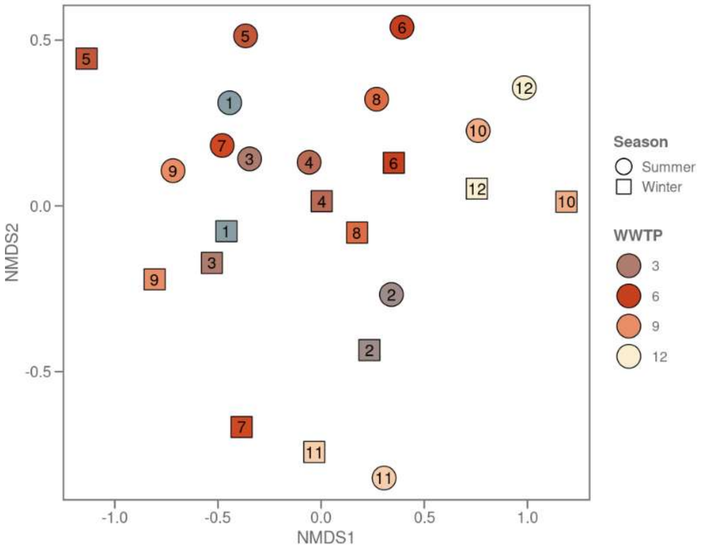

Supplementary Figure 2 – NMDS figure of the beta-diversity based on the Bray-Curtis dissimilarity of the microbial composition of the digestate samples.

Script for in silico primer testing in R

Scripts are shared on the public github link (https://github.com/jwvlandu/SeasonalVariationInAD)

```
> # Read the FASTA file that contains the 16S sequences of the isolates
> FASTA <- Biostrings::readDNAStringSet(filepath = "/Taxonomies/DADA2tax/DADA2_file_MiDAS_4.8.1.fa", format = "fasta") # Is from class 'DNAStringSet'
>
>
> # Check how many sequences
> length(FASTA) # paste(FASTA)
[1] 90164
> FASTA@ranges@NAMES <- paste0(FASTA@ranges@NAMES, 1:length(FASTA))
> FASTA_Archaea <- FASTA[grep("Archaea", FASTA@ranges@NAMES), ]
> FASTA_Archaea_methano <- FASTA_Archaea[grep("Methano", FASTA_Archaea@ranges@NAMES), ]
>
> # select for only microalgae/chloroplast <- how to?
>
> # Illumina primers (LGC)
> FPrimer <- Biostrings::DNAString("CCTACGGGNGGCWGCAG") # 341F
> RPrimer <- Biostrings::DNAString("GACTACHVGGGTATCTAAKCC") # 785Rmod
>
>
> # Write results to a dataframe to export later
> ToCSV <- data.frame(matrix(ncol = 0, nrow = length(FASTA)))
> ToCSV$IsolateID <- FASTA@ranges@NAMES
> ToCSV$TotalLength <- FASTA@ranges@width
>
>
> # Check for presence of the illumina primers ('vmatchpattern' for multiple sequences, 'matchpattern' for 1 sequence)
> # Forward
```

```
> MatchesForward1 <- Biostrings::vmatchPattern(pattern = FPrimer,
+                                 subject = FASTA,
+                                 fixed = FALSE,
+                               max.mismatch = 0) # fixed = FALSE: an IUPAC ambiguity code in the pattern can match any letter in the subject that is associated with the code, and vice versa
>
> MatchOrNot <- elementNROWS(MatchesForward1) #Contains a 1 in case of a match and a 0 in case of a non-match
> k <- as.data.frame(MatchOrNot)
> k$nr <- rownames(k)
> h <- k[k$MatchOrNot>0,]
> # double
> h$nr1 <- 1:nrow(h) # double
>
> MatchesForward2 <- unlist(MatchesForward1)
> MatchesForward <- MatchesForward2 # remove double
>
> ExactMatchesForward1 <- Biostrings::subseq(FASTA[as.logical(MatchOrNot)],
+                                start = MatchesForward@start,
+                                end = MatchesForward@start + length(FPrimer)-1)
>
> ExactMatchesForward <- as.data.frame(ExactMatchesForward1)
> ExactMatchesForward$names <- ExactMatchesForward1@ranges@NAMES
>
> # Archaea that have an exact match with the forward primer
> ExactMatchesForward_Archaea <- ExactMatchesForward[grep("Archae", ExactMatchesForward$names), ]
>
>
> ToCSV$ExactForwardMatch[match(ExactMatchesForward$names, ToCSV$IsolateID)] <- ExactMatchesForward$x # Save to the dataframe to export
> # Not for all sequences a match was found. Maybe the sequence was accidentaly in the reverse complement format?
> if (sum(MatchOrNot) < length(FASTA)){
```

```
+  MatchesForward_ <- Biostrings::vmatchPattern(pattern = FPrimer,
+                                subject = reverseComplement(FASTA[!as.logical(MatchOrNot)]),
+                                fixed = FALSE, max.mismatch = 0) # fixed = FALSE: an IUPAC ambiguity code in the pattern can match any letter in the subject that is associated with the code, and vice versa
+  MatchOrNot_ <- elementNROWS(MatchesForward_) # No
+ }
>
> sum( MatchOrNot_) # This is zero meaning none are in the reverse complement format
[1] 0
> # Maybe 1 base mismatch?
> if (sum(MatchOrNot) < length(FASTA)){
+    MatchesForward_ <- Biostrings::vmatchPattern(pattern = FPrimer, subject = FASTA[!as.logical(MatchOrNot)], fixed = FALSE, max.mismatch = 1)
+  MatchOrNot_ <- elementNROWS(MatchesForward_) # Yes
+
+  if(sum(MatchOrNot_) > 0){
+   ExactMatchesForward_1 <- Biostrings::subseq(FASTA[!is.na(match(FASTA@ranges@NAMES, MatchesForward_@NAMES))], start = unlist(startIndex(MatchesForward_)), end = unlist(endIndex(MatchesForward_)))
+   ExactMatchesForward_ <- as.data.frame(ExactMatchesForward_1)
+   ExactMatchesForward_$names <- ExactMatchesForward_1@ranges@NAMES
+    ToCSV$ExactForwardMatch[match(ExactMatchesForward_$names, ToCSV$IsolateID)] <- ExactMatchesForward_$x
+  }
+ }
>
>    ExactMatchesForward_Archaea <- ExactMatchesForward_[grep("Archae", ExactMatchesForward_$names), ]
> # Reverse
> MatchesReverse <- Biostrings::vmatchPattern(pattern = RPrimer, subject = reverseComplement(FASTA), fixed = FALSE, max.mismatch = 0)
> MatchOrNot <- elementNROWS(MatchesReverse)
> k <- as.data.frame(MatchOrNot)
> k$nr <- rownames(k)
> h <- k[k$MatchOrNot>0,]
```

```
> #h[h$nr == 1166,] # double 909

> #h[h$nr == 1716,] # double 1361

> h$nr1 <- 1:nrow(h) # double

>

> MatchesReverse <- unlist(MatchesReverse)

> #MatchesReverse <- MatchesReverse[c(-909,-1361),]

> ExactMatchesReverse1 <- Biostrings::subseq(reverseComplement(FASTA[as.logical(MatchOrNot)]), start = MatchesReverse@start, end = MatchesReverse@start + length(RPrimer) - 1)

>

> ExactMatchesReverse <- as.data.frame(ExactMatchesReverse1)

> ExactMatchesReverse$names <- ExactMatchesReverse1@ranges@NAMES

>

> ExactMatchesReverse_Archaea <- ExactMatchesReverse[grep("Archae", ExactMatchesReverse$names), ]

>

> ToCSV$ExactReverseMatch[match(ExactMatchesReverse$names, ToCSV$IsolateID)] <- ExactMatchesReverse$x # Save to the dataframe to export

> # Try for reverse complement

> if (sum(MatchOrNot) < length(FASTA)){

+ MatchesReverse_ <- Biostrings::vmatchPattern(pattern = RPrimer, subject = reverseComplement(FASTA[!as.logical(MatchOrNot)]), fixed = FALSE, max.mismatch = 0)

+  MatchOrNot_ <- elementNROWS(MatchesReverse_) # No

+ }

>

>

> # Try for 1 mismatch

> if (sum(MatchOrNot) < length(FASTA)){

+ MatchesReverse_ <- Biostrings::vmatchPattern(pattern = RPrimer, subject = FASTA[!as.logical(MatchOrNot)], fixed = FALSE, max.mismatch = 1)

+  MatchOrNot_ <- elementNROWS(MatchesReverse_) # No

+ }

>

> sum(MatchOrNot_) # no extra matches due to allowing of 1 mismatch

[1] 0
```

```
> # How many sequences are covered?
> TotalCovered <- 0
>
> for (i in 1:length(FASTA)){
+   if (!is.na(ToCSV$ExactForwardMatch[i]) & !is.na(ToCSV$ExactReverseMatch[i])) {
+     TotalCovered <- TotalCovered +1
+   }
+ }
> Total <- length(FASTA)
>
> PercentageCovered <- TotalCovered/Total*100
> PercentageCovered
[1] 92.71771
```

ExactMatchesReverse_Archaea = ExactMatchesForward_Archaea = 111 species
